# Comparison of Vaccine Platforms for Machupo Virus

**DOI:** 10.3390/vaccines14040315

**Published:** 2026-03-31

**Authors:** Rachel Erickson, Hiromi Muramatsu, Sachchidanand Tiwari, Sowmya Sriram, Fernanda Caroline Coirada, Norbert Pardi, Paul Bates

**Affiliations:** Department of Microbiology, Perelman School of Medicine, University of Pennsylvania, Philadelphia, PA 19104, USA; rachel.erickson@pennmedicine.upenn.edu (R.E.); hiromim@pennmedicine.upenn.edu (H.M.); sachchidanand.tiwari@pennmedicine.upenn.edu (S.T.); sowmya.sriram@pennmedicine.upenn.edu (S.S.); fernanda.coirada@pennmedicine.upenn.edu (F.C.C.); pnorbert@pennmedicine.upenn.edu (N.P.)

**Keywords:** mRNA vaccine, Machupo virus, Junín virus, recombinant VSV, mRNA virus-like particle, humoral response, cellular response

## Abstract

Background/Objectives: Pathogenic mammarenaviruses cause severe hemorrhagic and neurologic disease in humans. Machupo virus (MACV), a New World (NW) mammarenavirus, causes Bolivian hemorrhagic fever in humans, and there are no approved vaccines. Methods: Here, we describe and compare the immunogenicity of three vaccines expressing the MACV glycoprotein complex (GPC) in C57BL/6 mice: a recombinant vesicular stomatitis virus (rVSV) and two different lipid nanoparticle (LNP)-encapsulated nucleoside-modified mRNA (mRNA-LNP) vaccines. The first mRNA-LNP vaccine, designated MACV mRNA, expresses the full-length MACV GPC. The second mRNA-LNP vaccine, called MACV VLP mRNA, encodes MACV GPC with appended sequences that induce the budding of virus-like particles (VLPs) with MACV GPC on the surface. This is the first description of any mRNA-LNP vaccine for MACV and the first comparison of mRNA and rVSVs as vaccine candidates for MACV. Results: We find that two doses of either MACV mRNA or MACV VLP mRNA are required for the induction of robust humoral and cellular immune responses including total MACV GPC IgG, neutralizing antibodies, cross-reactive antibodies that bind the related Junín virus GPC, and MACV-specific T-cell responses. To further investigate vaccination strategies for MACV, we also evaluated a heterologous prime-boost regimen involving the MACV mRNA vaccine coupled with the rVSV-based MACV vaccine. We find that the highest levels of MACV GPC-specific IgG and neutralizing titers were achieved when heterologous mRNA and rVSV prime-boost regimens were employed. Conclusions: These results elucidate differences in the immune response to different vaccine platforms for MACV and can inform future vaccine development for NW arenaviruses.

## 1. Introduction

Human infection by mammarenaviruses (genus *Mammarenavirus*, family *Arenaviridae*) can lead to the development of severe viral hemorrhagic fever. Mammarenaviruses are classified into Old World (OW) and New World (NW) arenaviruses based on geographic distribution. OW arenaviruses are mostly found in Africa, whereas NW arenaviruses are found throughout the Americas. Pathogenic OW arenaviruses include Lassa virus (LASV, West Africa) and Lujo virus (Zambia), while lymphocytic choriomeningitis virus (LCMV) is found globally [1]. Pathogenic NW arenaviruses and the countries in which they are found include Machupo virus (MACV, Bolivia), Junín virus (JUNV, Argentina), Guanarito virus (GTOV, Venezuela), Chapare virus (CHAPV, Bolivia), Sabia virus (SABV, Brazil), and Whitewater Arroyo virus (WWAV, United States) [2]. The majority of the known hosts for mammarenaviruses are rodents with each virus highly adapted to a specific rodent species. The reservoir for MACV is the large vesper mouse, *Calomys callosus* [3,4]. Humans are most commonly infected by contact with an infected rodent host, its urine or fecal matter, the inhalation of dust with virus particles, or eating food containing remnants of the virus. Mammarenavirus transmission can also occur via human-to-human contact [5], and nosocomial transmission is documented [6,7].

MACV is the causative agent of Bolivian hemorrhagic fever (BHF) and is endemic in rural areas of northern Bolivia [8]. The mortality rate of BHF is approximately 30%. There are currently no FDA-approved treatments or vaccines for MACV infections. Due to its virulence, transmissibility, and threat to public health, MACV is classified by the NIH as a Category A priority pathogen, which is a designation that highlights the critical need for vaccine development [8].

Despite the threat to public health, there is currently only one vaccine approved for use in humans for any of the arenaviruses; Candid#1 is a live attenuated vaccine for JUNV. However, it is only approved for limited populations in Argentina where JUNV is endemic, and concerns about reversion to virulence remain [9]. Aside from Candid#1, the most advanced arenaviral vaccines in clinical trials are for the OW virus Lassa. These include a recombinant vesicular stomatitis virus (rVSV) vectored vaccine rVSVΔG-LASV-GPC [10,11], an adenoviral vectored vaccine ChAdOx1 Lassa [12], and LASSARAB, an attenuated rabies vectored vaccine that is subsequently inactivated to make the vaccine candidate [13]. Few MACV-specific vaccines are in development; platforms such as live attenuated vaccines [14,15], recombinant virus [16], and an alphavirus RNA replicon vector vaccine [17] have been tested. Although these vaccines show promise in animal models, they have not been tested in humans. Additionally, an ideal MACV vaccine would be one that protects against multiple other related NW mammarenaviruses such as JUNV [18].

Mammarenaviruses express a single viral surface glycoprotein complex (GPC) which is required for entry of the virus into host cells and is the target of neutralizing antibodies. Most mammarenaviral vaccines focus on developing a humoral immune response to the glycoprotein. The MACV glycoprotein precursor is cleaved by cellular proteases into a stable signal peptide, GP1, and GP2. GP1 is the surface glycoprotein and GP2 mediates viral fusion [19]. Data show that anti-MACV GPC sera is sufficient for protection from lethal MACV challenge in the established Hartley guinea pig model [20]. In humans, convalescent plasma has been investigated as a therapeutic for NW arenaviruses: a double-blind placebo-controlled study determined that the transfer of immune plasma significantly reduced the case fatality of JUNV [21]. The antibody titer in transferred serum correlated with survival from JUNV [22]. Serum transfer has also been used sporadically to treat cases of MACV infection; however, there is no established dose or protocol that is consistently protective [23]. These interventions support the importance of antibodies in controlling NW arenavirus infections, although the exact correlates of protection are not defined. Therefore, the characterization of antibody responses induced by infection and potential vaccine candidates is important.

Vesicular stomatitis virus (VSV) is a livestock pathogen; seroprevalence in humans is low, and infection rarely causes severe disease [24,25]. VSV can be engineered to express and incorporate foreign glycoproteins onto the surface of its virions to produce recombinant VSV (rVSV). These rVSVs have been evaluated as vaccines for a variety of pathogens as they can induce protective humoral and cellular responses [24,26,27,28,29,30,31]. The first FDA and WHO-approved rVSV vaccine, Ervebo, is a single-dose, live-attenuated vaccine for Ebola virus (EBOV). This vaccine has been successfully distributed to hundreds of thousands of individuals to combat recent EBOV outbreaks in Africa [28,32,33]. rVSV-based vaccines induce type 1 immune responses, which are ideal for the elimination of intracellular pathogens such as viruses [34,35,36,37]. Furthermore, immune responses to rVSV vaccines are very durable, and studies have reported T-cell and B-cell responses up to 5 years after vaccination with Ervebo [38,39]. VSV vaccines have been shown to provide rapid protection with studies showing some protection as early as three days post-vaccination [31,40].

Lipid nanoparticle (LNP)-encapsulated nucleoside-modified mRNA (mRNA-LNP) vaccines are a promising technology that have been used extensively not only in preclinical studies but also in the human population in recent years [41,42]. mRNA vaccines consist of an antigen-encoding in vitro transcribed mRNA encapsulated in LNPs that facilitates the delivery and subsequent expression of the vaccine antigen in the immunized organism, and it also acts as a potent adjuvant [43]. This platform allows for the rapid design and production of vaccines for emerging pathogens [42]. During the recent COVID-19 pandemic, mRNA vaccines for SARS-CoV-2 were successfully used to control infections and disease [41]. Data suggest that multiple doses of mRNA vaccines are necessary for optimal protection in humans. Overall, the mRNA platform is a powerful tool and potential vaccine candidate that should be studied in the context of other priority pathogens such as MACV and other NW arenaviruses.

Efforts to modify the existing mRNA vaccine platform to elicit a more robust immune response are also promising. A recently published technology alters traditional mRNA vaccines to induce the production of self-assembling enveloped virus-like particles (VLPs) [44]. In this system, mRNA encodes the viral glycoprotein with an endocytosis prevention motif (EPM) and an ESCRT and ALIX binding region (EABR) inserted into the cytoplasmic tail, which recruits cellular ESCRT proteins to induce VLP budding from cells. This allows for the presentation of the viral glycoprotein on cell surfaces and in VLPs, which is similar to the presentation of the antigen during natural infection. Hoffman et al. showed that an mRNA-LNP-encoding SARS-CoV-2 spike with EABR elicited superior neutralizing and higher overall antibody responses in mice compared to a conventional spike-encoding mRNA vaccine. The SARS-CoV-2 spike-EABR VLP construct also showed increased breadth as it elicited higher levels of neutralizing antibodies against SARS-CoV-2 variants [44].

We have developed an mRNA-LNP vaccine expressing the full-length MACV GPC (referred to here as MACV mRNA) as well as an mRNA-LNP expressing a MACV GPC EPM EABR that induces the formation of VLPs (referred to here as MACV VLP mRNA). These two mRNA vaccines were compared for the induction of total MACV GPC IgG and neutralizing antibodies as well as for the production of cross-reactive antibodies that bind and neutralize JUNV GPC. Furthermore, we measured cellular responses to vaccination with either MACV mRNA or MACV VLP mRNA. We find that two doses of 3 μg of MACV mRNA vaccine elicit robust humoral and cellular immune responses in C57BL/6 mice, whereas a single dose of 5 μg did not elicit a strong immune response. Two doses of the MACV VLP mRNA vaccine also elicits a strong humoral and cellular immune response with higher levels of MACV GPC-specific IgG and neutralizing antibodies and increased cross-reactive antibodies compared with MACV mRNA.

In an effort to develop vaccination strategies that provide protection against MACV, we also evaluated a heterologous prime-boost regimen involving MACV mRNA vaccines coupled with an rVSV-based MACV vaccine. Previously published data suggest the utility of combining vaccine platforms to elicit a more robust or favorable or protective response than each individual vaccine alone [45]. Many of these studies, however, focus on COVID-19 vaccines; [46] while informative, the conclusions from these studies are not necessarily broadly applicable to other viruses. In this study, the highest levels of MACV GPC-specific IgG and neutralizing titers were achieved when heterologous mRNA and rVSV prime-boost regimens were employed. Together, these results highlight differences in the immune response to different vaccine platforms for MACV and can help inform future vaccine development for NW arenaviruses.

## 2. Materials and Methods

### 2.1. Cell Lines and Plasmids

ATCC verified and mycoplasma free 293T and Vero E6 cells were maintained in DMEM (Corning, Corning, NY, USA; #10-013-CV) containing 10% CCS (HyClone, Logan, UT, USA; #SH30087.03). Cells were passaged every 2–3 days. A full-length, codon-optimized MACV GPC sequence was obtained as a gBlock (Integrated DNA Technologies (IDT), Coralville, IA, USA) and cloned into a pCG1 expression vector to create pCG1-MACV GPC. The EPM-EABR sequence was obtained as a gBlock as well and then cloned into the pCG1 MACV GPC expression plasmid at various positions in the MACV GPC cytoplasmic tail to create MACV GPC (full-length) + EPM EABR, MACV Δ13 + EPM EABR, and MACV Δ25 + EPM EABR. The EPM-EABR sequence was as follows: EPM: ALPGNPDHREMGETLPEEVGEYRQPSGGSVPVSPGPPSGLEPTSSSPY, GS linker: GSGSGS, EABR: FNSSINNIHEMEIQLKDALEKNQQWLVYDQQREVYVKGLLAKIFELEKKTETAAHSLP [44].

### 2.2. SDS-PAGE and Western Blot

Vero E6 cells were transfected with Lipofectamine3000 (ThermoFisher, Waltham, MA, USA; L3000015) according to the manufacturer’s protocol. For mRNA transfection, TransIt mRNA (MirusBio, Madison, WI, USA; MIR 2225) was used according to the manufacturer’s protocol. At 48 h post-infection, cells were collected from the plate, pelleted by centrifugation at 1250× *g* for 10 min, and then washed 1× with PBS. The cell pellet was resuspended in RIPA buffer + complete protease inhibitor (Roche, Indianapolis, IN, USA) and rocked at 4 °C for 1 h. The samples were spun at 21,300× *g* at 4 °C for 10 min, and the supernatant was collected as cell lysate.

Cell lysate from transfected cells was mixed with 6x loading dye and boiled at 95 °C for 5 min. Proteins were examined using SDS-PAGE. For Western blotting, proteins were separated and transferred onto a poly (vinylidene fluoride) (PVDF) membrane, blocked with blocking buffer (5% NFDM in TBS + 0.5% Tween 20), and incubated with serum from MACV mRNA immunized mice (1:1000 in blocking buffer) at 4 °C overnight on a plate rocker. This was followed by incubation for 1 h with anti-mouse HRP-conjugated secondary antibody, the addition of substrate (Invitrogen, Carlsbad, CA, USA; # 31430), and imaging on an Amersham Imager 600 (GE Healthcare, Chicago, IL, USA).

### 2.3. Animal Care and Use

For mouse studies, 8-week-old female C57BL/6 mice (JAX, Bar Harbor, ME, USA; stock #000664) were used. Mice were maintained according to the Guide for the Care and Use of Laboratory Animals. All animal work was approved by the University of Pennsylvania institutional animal care and use committees (IACUC). All procedures were performed on animals after they were anesthetized by trained personnel. All efforts were made to minimize the pain and suffering of animals.

### 2.4. Production of mRNA-LNP Vaccines

mRNAs were generated as previously described [47]. Briefly, the codon-optimized sequences for MACV GPC, MACV GPC EPM EABR, and firefly luciferase were synthesized (Genscript, Piscataway, NJ, USA) and cloned into an mRNA production plasmid. MEGAscript T7-driven in vitro transcription reactions (Ambion, Austin, TX, USA, #AM1334) using linearized plasmid templates were performed to generate mRNAs with a 101 nucleotide long poly(A) tail.

The capping of mRNAs was performed in concert with transcription through the addition of a trinucleotide cap1 analog CleanCap, and m1Ψ-5′-triphosphate (TriLink, San Diego, CA, USA) was incorporated into the reaction instead of uridine-5′-triphosphate (UTP). The cellulose-based purification of mRNAs was performed, and the integrity of the purified mRNAs was evaluated on an agarose gel before storage at −20 °C.

Cellulose-purified mRNAs were encapsulated in LNPs using a self-assembly process where an aqueous solution of mRNAs at pH 4.0 is rapidly mixed with a solution of lipids dissolved in ethanol. In brief, lipids were dissolved in ethanol at a molar ratio of 50:38.5:10:1.5 (SM102:Cholesterol:DSPC:DMG-PEG), and mRNA was diluted in citrate buffer (50 mM, pH-4) at a concentration of 129 µg/mL. Both phases were mixed at a volumetric ratio of 1:3 (ethanol: citrate buffer) using a microfluidic mixing device (NanoAssembler Ignite, Precision Nanosystems, Vancouver, BC, Canada). The LNPs were then dialyzed against 1× PBS buffer in a 12–14 kDa dialysis membrane (Fisher Scientific, Waltham, MA, USA; Cat#08700158) for 2 h. The LNP formulations were characterized for their size, polydispersity index and zeta potential using Zetasizer Pro ZS (Malvern Panalytical, Westborough, MA, USA). The mRNA encapsulation efficiency in the LNPs was quantified using the Quant-it RiboGreen assay (ThermoFisher Scientific).

The mRNA-LNP vaccine encoding influenza virus HA (A/Michigan/45/2015 H1) was shared by Scott Hensley’s lab at the University of Pennsylvania.

### 2.5. Immunization and Blood Collection

mRNA LNPs were diluted in sterile PBS just prior to inoculation. The 8-week-old female C57BL/6 mice (JAX, stock #000664) were anesthetized with isoflurane and immunized intramuscularly (i.m.) in the hind leg with 1–10 μg of mRNA LNP (50 μL total volume).

rVSV-MACV was diluted in sterile PBS just prior to inoculation. The 8-week-old female C57BL/6 mice (JAX, stock #000664) were anesthetized with isoflurane and immunized intraperitoneally (i.p.) with rVSV (150 μL total volume). We anticipated a low replication of VSV-MACV in mice due to the receptor–glycoprotein mismatch [48] and chose the vaccine dose to compensate and allow for VSV replication [49]. To further address this issue and allow efficient VSV replication, 0.5 μg/mouse of an anti-interferon-alpha/beta receptor (anti-IFNAR-1) blocking antibody (Clone MAR1-5A3) (Leinco Technologies Inc., Fenton, MO, USA; #I-1188 or Biolegend, San Diego, CA, USA; #127321) was administered 1 day before and 1 day after vaccination.

Blood was collected via the submandibular route using Goldenrod lancets 5 mm (Medipoint, Mineola, NY, USA). Serum was separated from blood by centrifugation at 7000× *g* for 30 min at 4°C in an Eppendorf 5424R centrifuge (Eppendorf, Enfield, CT, USA). EDTA was added to each sample for a final concentration of 5 mM. Serum was heat inactivated by incubating at 53°C for 30 min and then centrifuged at 24,400× *g* for 20 min. Serum samples were stored at −20 °C.

### 2.6. Production of Recombinant Proteins

The MACV GPC sequence was cloned from pCAGGS-MACV GPC into a Sport6 expression vector. This vector includes a trimerization domain and a 6His tag. Expi293 cells (Gibco, Waltham, MA, USA; #A14527) grown in Expi293 Expression media (Gibco, #A1435101) were transfected with the Sport6 MACV GPC plasmid using PEI Max. The supernatant was collected 4 days post-transfection and purified by nickel–nitrilotriacetic acid resin (Qiagen, Germantown, MD, USA; #30210) according to the manufacturers’ protocol. The eluted protein was concentrated and the buffer was exchanged into PBS using an Amicon Ultra-15 Centrifugal Filter with a 30 kDa MWCO (Millipore, Burlington, MA, USA; #UFC903024). Thet purified protein was quantified using a Pierce BCA protein assay kit (Thermo Scientific, 23225); then, it was aliquoted and stored at −80 °C. The recombinant influenza HA (Michigan/15) protein was obtained from Scott Hensley’s lab at the University of Pennsylvania.

### 2.7. Enzyme-Linked Immunosorbent Assay (ELISA)

Immulon 2HB (Thermo Scientific, Waltham, MA, USA; #3455) plates were coated with 50 μL of 1 µg/mL of purified MACV GPC or influenza virus HA in PBS at 4 °C overnight. The next day, ELISA plates were washed with PBS containing 0.1% Tween-20 (PBS-T) and blocked for an hour at room temperature on a plate shaker with 3% milk in PBS-T. The mouse serum was diluted in 1% milk in PBS-T and then serially diluted 2-fold. Plates were incubated with diluted mouse serum for 2 h at room temperature. Plates were washed with PBS-T before adding a goat anti-mouse IgG (H + L) secondary antibody (HRP-conjugated) (Invitrogen, Carlsbad, CA, USA; #31430), which was diluted in 1% PBS-T at 1:2000. Plates were incubated at room temperature for one hour. KPL SureBlue TMB 1 component substrate (Sera Care, Milford, MA, USA; #52-00-01) was then added to plates and quenched after 5 min with 250 mM HCl. Absorbance at 450 nm was immediately read on a SpectraMax 190 microplate reader (Molecular Devices, San Jose, CA, USA).

The endpoint titer was determined from interpolated values based on an anti-His antibody standard (Developmental Studies Hybridoma Bank (DSHB), Iowa City, IA, USA; N144/14) run on each plate. Samples lacking absorbance at our lowest dilution of 1:20 were assigned a titer of 1 to indicate titer below the limit of detection. The mean endpoint titers for each sample were reported based on measurements from at least two technical replicates.

### 2.8. Focus Reduction Neutralization Test (FRNT)

To produce VSVΔG-RFP MACV pseudotypes, HEK 293T cells were seeded at 5 × 10^6^ cells per 10 cm dish, incubated for 24 h, and transfected using calcium phosphate with 25 μg of pCG1 MACV plasmid. Then, 24 h post-transfection, cells were infected for 2 h with VSV-G pseudotyped VSVΔG-RFP at an MOI of ∼1–3. VSVΔG-RFP MACV pseudotypes were harvested by collecting the culture media 28–30 h after infection. These supernatants were clarified by centrifugation twice at 6000 g and stored at −80 °C.

For the neutralization assay, Vero E6 cells were seeded at a density of 2.5 × 10^4^ cells/well in 100 μL in a 96-well collagen-coated plate. The VSVΔG-RFP MACV pseudotype virus (100–300 focus forming units/well) was mixed with serum samples from a serial two-fold dilution and incubated for 1 h at 37 °C. To neutralize any potential VSV-G carryover virus, mouse anti-VSV Indiana G antibody (1E9F9) was also added at a concentration of 600 ng/mL (Absolute Antibody, Newark, CA, USA Ab01402-2.0), and the VeroE6 cell culture media was replaced with this serum–virus mixture. After 21–22 h, VeroE6 cells were washed and fixed with 4% paraformaldehyde before visualization on an S6 FluoroSpot Analyzer (CTL, Shaker Heights, OH, USA). The individual infected foci were enumerated, and the values were compared to control wells without antibody. The focus reduction neutralization titer 50% (FRNT50) was measured as the greatest serum dilution at which the focus count was reduced by at least 50% relative to the control cells that were infected in the absence of serum. The geometric mean FRNT50 titers for each sample were reported based on readings from at least two technical replicates.

### 2.9. Virus-like Particles (VLP) Production

Expi293 cells (Gibco, #A14527) grown in Expi293 Expression media (Gibco, #A1435101) were transfected with the pCG1 MACV EPM EABR plasmid using PEI Max (Kyfora Bio, Horsham, PA, USA; 24765). The cells and supernatant were collected 72 h post-transfection. Cell lysates were prepared as described above. The supernatant was filtered with a 0.45 um filter (Denville Scientific Inc., Holliston, MA, USA; 1159T83) and then concentrated via ultracentrifugation. VLPs were spun on a 20% sucrose cushion for 2 h at 96,281× *g* in an Optima XPN-80 ultracentrifuge (Beckman Coulter, Indianapolis, IN, USA) with an SW 32 Ti rotor. The VLP pellet was resuspended in RIPA for further analysis via Western blotting.

### 2.10. Enzyme-Linked Immunospot (ELISpot)

A peptide pool spanning the entire MACV GPC sequence comprising 49 peptides spanning GP1 and 48 peptides spanning GP2 was obtained from Genscript as 15-mers with 10 amino acid overlaps.

Enzyme-linked Immunospot (ELISpot) plates (MilliporeSigma MultiScreen 96-Well Assay Plates for ELISpot Assays- MAIPS4510) were activated with 30% ethanol and then coated with Mouse IFN-γ capture antibody (BD Biosciences, San Diego, CA, USA; #551881) at 4 °C overnight. The next day, plates were washed and blocked with RPMI + 10% FBS for 2 h at room temperature. Splenocytes from immunized mice were added to the plate at 300k cells/well along with VSV N or MACV GPC peptides at 1 μg/mL and recombinant IL-2. The plate was incubated at 37 °C, 5% CO_2_ for 20 h. After incubation, the plate was washed with PBS-T (1× PBS + 0.05% Tween 20), and the biotinylated anti-mouse IFN-γ detection antibody (BD Biosciences, San Diego, CA, USA; #551881) was added to wells for 2 h. Plates were washed again, and then streptavidin–HRP (BD Biosciences, San Diego, CA, USA; #557630) was added for 1 h. Plates were washed with PBS, and freshly prepared AEC substrate solution (BD Biosciences, San Diego, CA, USA; #B551951) was added to the wells. Wells were monitored for 10–60 min for the appearance of spots; then, the reaction was stopped by running the plate under tap water. After the plate was dried, it was scanned with the S6 FluoroSpot Analyzer (CTL, Shaker Heights, OH, USA). Individual spots were enumerated and compared to control wells without stimulus. The data were reported as the average of two technical replicates.

### 2.11. VSV Launch

The rVSV-MACV genome was created by cloning the full-length, codon-optimized MACV GPC gene (IDT) into pVSV eGFP RABVG plasmid (Addgene, Watertown, MA, USA). The rVSV-MACV was generated by inserting MACV GPC into the pVSV eGFP RABV G backbone between M and L, replacing RABV G. This vector also contains eGFP as an additional ORF upstream of N. VSV launch plasmids were also obtained from Addgene. After generation, vectors were sequenced by Eurofins’ complete plasmid sequencing service.

pVSV eGFP RABV-G was a gift from Connie Cepko [50] (Addgene plasmid # 31833; http://n2t.net/addgene:31833 (accessed on 29 March 2023); RRID:Addgene_31833). pCAG-VSVN was a gift from Ian Wickersham (Addgene plasmid # 64087; http://n2t.net/addgene:64087 (accessed on 29 March 2023); RRID:Addgene_64087). pCAG-VSVP was a gift from Ian Wickersham (Addgene plasmid # 64088; http://n2t.net/addgene:64088 (accessed on 29 March 2023); RRID:Addgene_64088). pCAG-VSVL was a gift from Ian Wickersham (Addgene plasmid # 64085; http://n2t.net/addgene:64085 (accessed on 29 March 2023); RRID:Addgene_64085). T7opt in pCAGGS was a gift from Benhur Lee [51] (Addgene plasmid # 65974; http://n2t.net/addgene:65974 (accessed on 29 March 2023); RRID:Addgene_65974).

Recombinant VSV viruses were launched as described previously [52]. Briefly, 293T cells were plated 24 h before transfection with Lipofectamine 3000 (Thermo Fisher Scientific, Waltham, MA, USA). The plasmids transfected were the VSV genome, VSV N, VSV P, VSV L and T7 opt polymerase at a 1:3:1:1:2 molar ration with a total of 2.7 μg DNA per well in a 6-well plate. The media was changed 18–24 h post-transfection. Cells were observed for eGFP expression and cytopathic effects, including cell rounding and death. Virus was collected 2–4 days post-transfection, cell debris was spun down at 1250× *g* for 10 min, and then the supernatant was passaged onto a monolayer of Vero E6 cells.

All recombinant virus stocks were grown in Vero E6 cells by infecting a confluent 15 cm plate at an MOI of 0.0001. The virus was collected 48 h post-infection, and the media was clarified by centrifugation at 1250× *g* for 10 min at 4 °C. The virus was then frozen at −80 °C until concentrated via ultracentrifugation. The virus was spun on a 20% sucrose cushion for 2 h at 96,281× *g* in an Optima XPN-80 ultracentrifuge (Beckman Coulter, Indianapolis, IN, USA) with an SW 32 Ti rotor. The virus pellet was resuspended in PBS, aliquoted, and stored at −80 °C.

### 2.12. Statistical Analyses

GraphPad Prism Version 10.6.1 was utilized for analysis, as defined in each figure legend.

## 3. Results

### 3.1. Characterization and Immunogenicity of the MACV mRNA Vaccine

**Figure 1:**
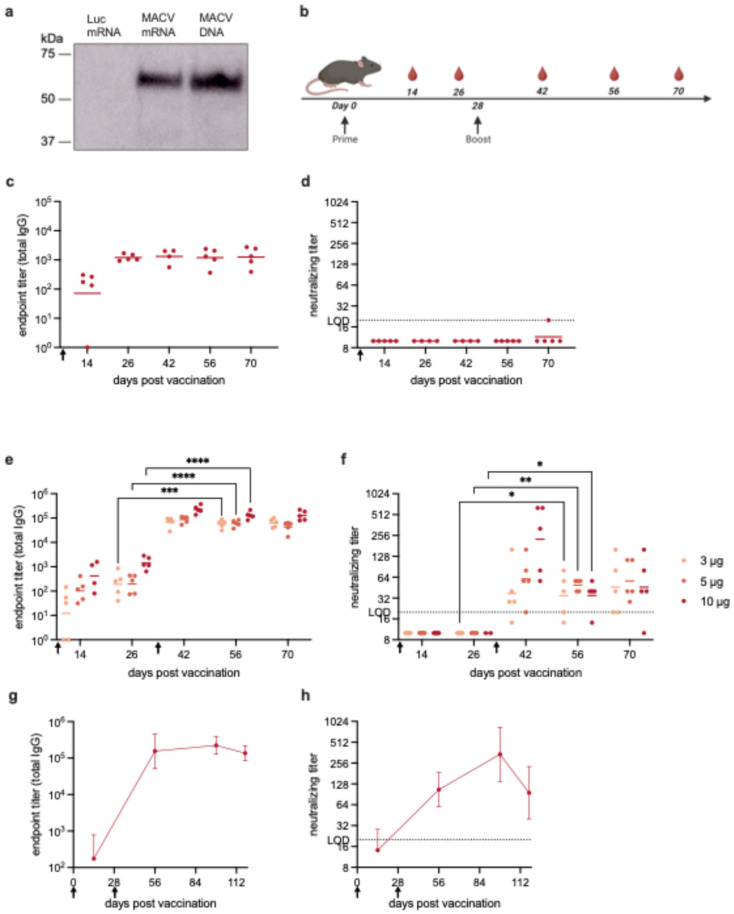
Characterization and immunogenicity of MACV mRNA vaccine. (**a**) Western blot showing the in vitro expression of transfected MACV mRNA. (**b**) Experimental timeline for immunization in C57BL/6 mice. Mice (*n* = 5 per group) were vaccinated with MACV mRNA on day 0 and boosted (if applicable) on day 28, which is indicated by black arrows. Red blood droplets indicate blood collection, which took place every 2 weeks for serum analysis of antibody titers. Total MACV-specific IgG titers (**c**) and neutralizing titers (**d**) in serum from mice vaccinated with a single dose of MACV mRNA (5 μg). Total MACV-specific IgG titers (**e**) and neutralizing titers (**f**) in serum from mice vaccinated with two doses of MACV mRNA (3, 5, or 10 μg). Symbols represent individual animals, and the line indicates the geometric mean of the group. Each point represents the mean (ELISA) or geometric mean (FRNT) from 2–3 independent ELISA or FRNT analyses of each serum sample. Longevity of total IgG (**g**) and neutralizing (**h**) antibody responses in mice that received two 10 μg doses of MACV mRNA. Data are shown as geometric mean ± standard deviation. LOD = limit of detection. Black arrows on the X-axis indicate the timing of prime and boost immunizations. (**e**,**f**). Statistical analyses were performed to compare the titer elicited by each dose before (on day 26) and after the boost (on day 56), as each are approximately 4 weeks post-immunization. Welch’s *t* test was performed when data met the assumption of normality; otherwise, the Mann–Whitney test was applied. (**g**,**h**) Statistical analyses were performed to compare different vaccination groups on individual days. One-way ANOVA with Tukey’s multiple comparisons test was performed when data met the assumption of normality; otherwise, the Kruskal–Wallis with Dunn’s multiple comparisons test was applied, * *p* < 0.05, ** *p* < 0.01, *** *p* < 0.001, **** *p* < 0.0001.

We developed an mRNA-LNP vaccine encoding the full-length MACV GPC, which is referred to here as MACV mRNA. Protein expression was confirmed by transfecting MACV mRNA into 293T cells and performing Western blot analysis on cell lysates (Figure 1a and Supplementary Figure S1). Parallel transfection with firefly luciferase (Luc)-encoding mRNA and a plasmid-encoding MACV GPC were included as negative and positive controls, respectively. To evaluate the immunogenicity of the MACV mRNA vaccine, C57BL/6 mice were immunized intramuscularly (i.m.) with 5 μg of mRNA-LNP. Groups of mice were also given 5 μg of Luc mRNA-LNP or PBS as negative controls. Serum was collected every 2 weeks post-vaccination for analysis of antibody responses (Figure 1b). Mice exhibited no signs of weight loss or illness over the course of the study.

Using a MACV GPC Enzyme-Linked Immunosorbent Assay (ELISA), we measured total IgG titers induced by the MACV mRNA vaccine. Fourteen days after a single mRNA vaccination, very low antibody titers were elicited. Titers rose slightly by 4 weeks post-vaccination but did not continue to increase over time (Figure 1c). To further explore the humoral immune response to vaccination, we measured neutralizing antibody titers via a focus reduction neutralization test of 50% (FRNT_50_). There were no measurable neutralizing antibodies at any time after the administration of a single vaccine dose (Figure 1d). Negative control sera from Luc mRNA-LNP- and PBS-immunized mice were run in parallel and exhibited no reactivity to MACV GPC via ELISA or FRNT.

To explore if an increased mRNA-LNP dose or a booster immunization would improve the humoral immune response, mice were immunized with 3, 5, or 10 μg of MACV mRNA on day 0. All mice received a second dose 28 days after priming that was identical to the first dose. Initial antibody titers were still low even at the highest dose of 10 μg mRNA-LNP. The administration of a boost increased the titers significantly, up to approximately 10^5^ for all doses of MACV mRNA (Figure 1e). The fold change in the IgG titer from 4 weeks post prime (day 26) to 4 weeks post boost (day 56) was 299.69 for the 3 μg group, 305.98 for the 5 μg group, and 91.05 for the 10 μg group. There appeared to be a modest dose response for the IgG titers with the 10 μg mRNA cohort having the highest titers.

Similarly, no neutralizing antibodies were detected after prime immunization, but following the boost, neutralizing antibodies developed. Neutralizing titers were between 32 and 64 for the 3 μg and 5 μg doses of MACV mRNA. These titers remained relatively constant through day 70 post-vaccination (6 weeks post-boost). For the 10 μg immunizations, the neutralizing antibody titer peaked on day 42 post-vaccination (2 weeks post-boost); then, it declined slightly and stabilized for the duration of the experiment (Figure 1f). To further examine the durability of the anti-MACV antibody responses, mice were immunized and boosted with 10 μg mRNA and then followed for 118 days. The total IgG antibody titers remained constant for at least 3 months after administration of the second dose (Figure 1g). There was some fluctuation in the neutralizing antibody titer over the course of the study, but overall, the neutralizing titers did not decline significantly (Figure 1h). These results show that two doses of MACV mRNA-LNP are required for the induction of a robust and durable antigen-specific humoral immune response in mice.

### 3.2. Investigating the Low Immunogenicity of the MACV mRNA Vaccine

Due to the low initial antibody titers and lack of neutralizing antibodies, we sought to verify that the MACV mRNA vaccine was not poorly expressed in vivo or exhibiting some sort of inhibitory effect on the antibody response to vaccination. Previous studies from our labs showed robust antibody responses with a single dose of mRNA vaccine for a variety of viral antigens [45,53,54].

**Figure 2:**
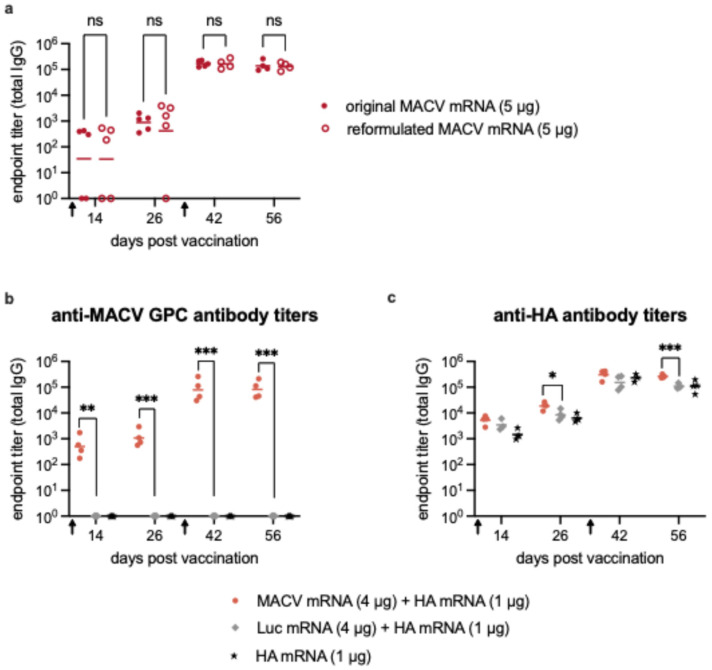
Investigating the low immunogenicity of the MACV mRNA vaccine. (**a**) Total MACV-specific IgG titers in serum from mice (*n* = 5 per group) vaccinated with 5 μg of the original MACV mRNA (solid red circles) or the reformulated MACV mRNA (open red circles). Total MACV-specific IgG titers (**b**) or HA-specific IgG titers (**c**) in serum from mice (*n* = 4 per group) co-vaccinated with MACV and HA mRNA (light red circles), Luc and HA mRNA (gray diamonds), or HA mRNA alone (black stars). Black arrows on the X-axis indicate the timing of prime and boost immunizations. Symbols represent individual animals, and the line indicates the geometric mean of the group. Each point represents the mean from 2–3 independent ELISA analyses of each serum sample. Statistical analyses were performed to compare the original and reformulated MACV mRNA groups (**a**) or MACV mRNA + HA mRNA and Luc mRNA + HA mRNA groups (**b**,**c**) on individual days. Welch’s *t* test was performed when data met the assumption of normality; otherwise, the Mann–Whitney test was applied, ns = not significant, * *p* < 0.05, ** *p* < 0.01, *** *p* < 0.001.

To ensure that the mRNA component of the vaccine was not responsible for the initially low titers, the nucleotide sequence encoding MACV GPC was modified. Initially, we utilized a codon-optimized sequence with high GC content to increase protein expression. An alternative mRNA corresponding to the original viral RNA sequence was synthesized, cloned and used to produce mRNA. This was then reformulated into a new MACV mRNA-LNP vaccine. Mice were immunized and boosted with 5 μg of either the original or the reformulated MACV mRNA as described previously. There was no difference between the total IgG titers elicited by the two vaccines (Figure 2a). As before, the initial anti-MACV titers before the boost were low and increased by approximately 200-fold upon boost.

We next hypothesized that the low anti-MACV titers could be because MACV GPC itself is inhibiting initial antibody development. To test this idea, we co-vaccinated mice with 4 μg of MACV mRNA and 1 μg of influenza virus hemagglutinin (HA) mRNA or 4 μg of Luc mRNA and 1 μg of HA mRNA. MACV-specific titers were similar to those obtained from previous experiments, demonstrating that the addition of HA mRNA did not alter the total anti-MACV IgG titers (Figure 2b). ELISA for anti-HA IgG demonstrated that co-immunization with MACV and HA mRNA did not inhibit the antibody response to HA and may have even had a slight stimulatory effect (Figure 2c). This is to be expected, as the LNP is a strong adjuvant; the LNP in the 4 μg of MACV mRNA will increase the HA antibody response induced by 1 μg HA mRNA-LNP. Although the amount of MACV mRNA used for immunization was four times higher than the amount of HA mRNA, the magnitude of the anti-MACV antibody response was not as robust as the anti-HA response. As observed previously following a boost, anti-MACV IgG titers increased (76.29 fold from day 26 to day 56) to levels slightly lower than the boosted anti-HA titers. Overall, these results suggest that it is an inherent feature of MACV GPC responsible for the low IgG and neutralizing antibody responses.

### 3.3. Immunogenicity of MACV Virus-like Particle mRNA Vaccine

**Figure 3:**
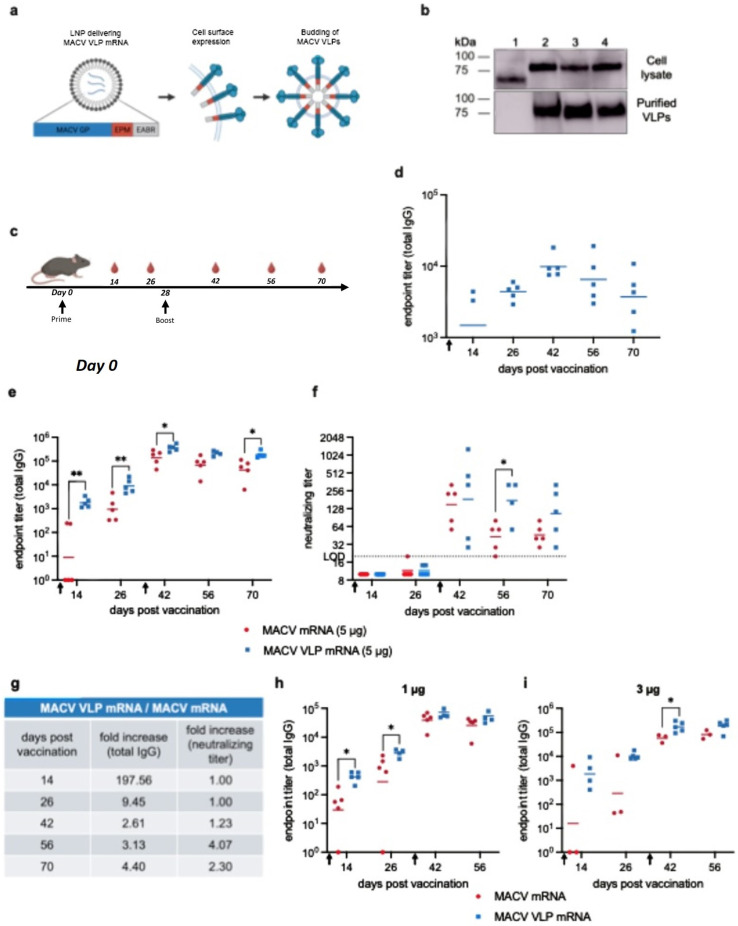
Characterization and immunogenicity of MACV VLP mRNA vaccine. (**a**) Cartoon illustrating how MACV VLP mRNA induces the budding of VLPs from the plasma membrane. mRNA encodes MACV GPC, an endocytosis prevention motif (EPM), and the ESCRT- and ALIX-binding region (EABR). (**b**) Expi293 cells were transfected with plasmids expressing 1. MACV GPC, 2. MACV GPC (full-length) + EPM EABR, 3. MACV Δ13 + EPM EABR, 4. MACV Δ25 + EPM EABR. Western blot analysis of cell lysate (top) or purified VLPs concentrated from cell supernatant via ultracentrifugation (bottom). (**c**) Experimental timeline for immunization in C57BL/6 mice. Mice (*n* = 5 per group) were vaccinated with MACV mRNA or MACV VLP mRNA on day 0 and boosted (if applicable) on day 28, as indicated by black arrows. Red blood droplets indicate blood collection which took place every 2 weeks for serum analysis of antibody titers. (**d**) Total MACV-specific IgG titers in serum from mice vaccinated with a single dose of MACV VLP mRNA (5 μg). Total MACV-specific IgG titers (**e**) and neutralizing titers (**f**) in serum from mice vaccinated with two 5 μg doses of MACV mRNA (red circles) or MACV VLP mRNA (blue squares). LOD = limit of detection. (**g**) Fold change in response to MACV VLP mRNA versus MACV mRNA at different time points post-vaccination. Total MACV GPC-specific IgG titers in serum from mice vaccinated with 1 μg (**h**) or 3 μg (**i**) of MACV mRNA (red circles) or MACV VLP mRNA (blue squares). Black arrows on the X-axis indicate the timing of prime and boost immunizations. Symbols represent individual animals, and the line indicates the geometric mean of the group. Each point represents the mean (ELISA) or geometric mean (FRNT) from 2–3 independent ELISA or FRNT analyses of each serum sample. (**e**,**f**,**h**,**i**) Statistical analyses were performed to compare MACV mRNA and MACV VLP mRNA groups on individual days. Welch’s *t* test was performed when data met the assumption of normality; otherwise, the Mann–Whitney test was applied, * *p* < 0.05, ** *p* < 0.01.

We sought to improve the immunogenicity of the conventional MACV mRNA vaccine by altering the mRNA to induce the production of self-assembling virus-like particles (VLPs) [44]. This is achieved by appending two sequences to the cytoplasmic tail of the encoded MACV GPC: an endocytosis prevention motif (EPM) and an ESCRT and ALIX-binding region (EABR) which recruits cellular ESCRT proteins to induce VLP budding from cells. The altered mRNA is encapsulated in LNPs as in traditional mRNA vaccines. Immunization with this altered mRNA-LNP results in an antigen that is not only expressed on the cell surface, but also in VLPs, similar to the presentation of the antigen during natural infection. Employing this approach created a vaccine that we called MACV VLP mRNA (Figure 3a).

We created DNA plasmids expressing the MACV GPC constructs that had EPM and EABR appended to the cytoplasmic tail. To evaluate if the MACV GPC cytoplasmic tail length affected the ability to form VLPs, we tested the full-length MACV GPC as well as two different truncations of the cytoplasmic tail (13 amino acids and 25 amino acids). All constructs expressed similarly in cell lysates as measured by Western blotting (Figure 3b). The addition of the EPM and EABR sequences to the MACV GPC protein resulted in a slightly higher molecular weight; therefore, the protein produced by the MACV VLP construct migrated more slowly upon gel analysis than MACV GPC alone. To verify that MACV VLPs were being produced, we collected the supernatant from transfected cells and then used ultracentrifugation to pellet and purify particles. There was no MACV GPC detected in the pelleted sample from the supernatant from cells expressing MACV GPC alone (lane 1). However, in the supernatant from cells transfected with MACV GPC + EPM EABR, MACV GPC was detected, indicating the presence of MACV VLPs (lane 2). The cytoplasmic tail truncation of MACV GPC did not seem to greatly affect the expression levels of the protein or the formation of VLPs (Figure 3b and Supplementary Figures S2 and S3). We chose to use the full-length MACV GPC sequence with EPM and EABR sequences in our vaccine, which is referred to hereafter as the MACV VLP mRNA vaccine.

To evaluate the immunogenicity of the MACV VLP mRNA vaccine, we immunized mice as described previously for MACV mRNA. In an initial experiment, mice received 5 μg of MACV VLP mRNA, and serum was collected for analysis of antibody titers (Figure 3c). Unlike MACV mRNA vaccination, a single dose of MACV VLP mRNA elicited modest antibody titers at day 14, which continued to increase until day 42 and then decreased slightly (Figure 3d). To directly compare the MACV mRNA and MACV VLP mRNA vaccines, mice were vaccinated with either MACV mRNA or MACV VLP mRNA and then boosted 28 days later (Figure 3c). The MACV VLP mRNA vaccine elicited significantly higher levels of antibodies than MACV mRNA, which were approximately 200-fold higher on day 14 and 9.45-fold higher on day 26. The difference between groups was not as pronounced following the boost, but titers from the MACV VLP mRNA-vaccinated mice were still about 2-fold higher (Figure 3e,g). This trend of superior response to MACV VLP mRNA is maintained at lower doses of vaccine (1 and 3 μg), again with larger differences between groups pre-boost, and more modest but still noticeable higher levels in the MACV VLP groups post-boost (Figure 3h,i). The MACV VLP mRNA vaccine exhibited a dose response trend with the 5 μg dose eliciting the highest levels of IgG titers (Figure 3e,h,i).

Unlike the increased IgG titers seen with the VLP mRNAs, the initial neutralizing antibody titers induced by the two vaccines were similar with little to no detectable neutralizing antibodies pre-boost (Figure 3f). After the boost, the VLP mRNA vaccine induced higher levels of neutralizing antibodies. Four and six weeks post boost, neutralizing antibodies in serum from mice immunized with MACV VLP were 4.07 and 2.30-fold higher, respectively, than from mice that received MACV mRNA (Figure 3f,g). Overall, the MACV VLP mRNA vaccine elicited higher levels of total IgG and neutralizing antibodies than MACV mRNA. However, both vaccines still require two doses to achieve a robust anti-MACV humoral response.

### 3.4. Breadth of Humoral Immune Response to Vaccination with MACV mRNA Vaccines

**Figure 4:**
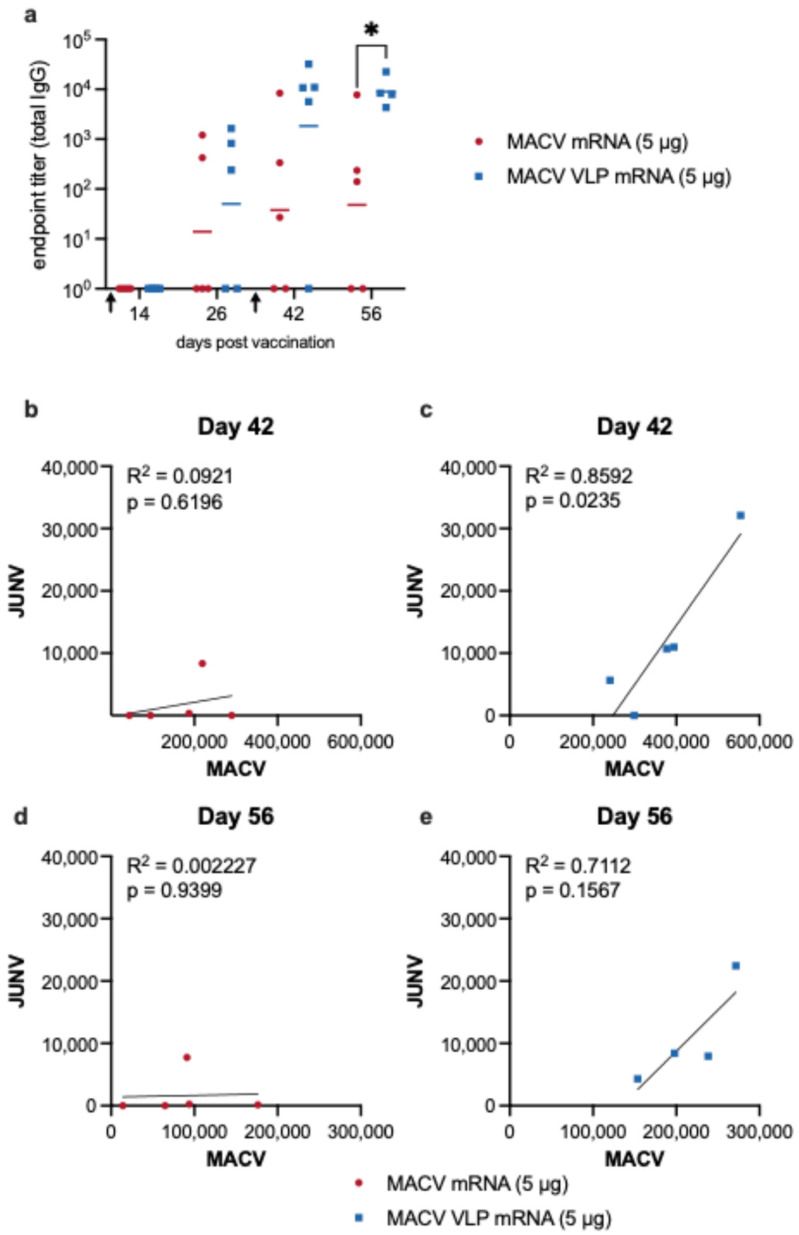
Breadth of humoral immune response to vaccination. (**a**) Total JUNV-specific IgG titers in serum from mice (*n* = 5 per group) vaccinated with two 5 μg doses of MACV mRNA (red circles) or MACV VLP mRNA (blue squares). Black arrows on the X-axis indicate the timing of prime and boost immunizations. Symbols represent individual animals, and the line indicates the geometric mean of the group. Each point represents the mean from 2–3 independent ELISA analyses of each serum sample. Statistical analyses were performed to compare MACV mRNA and MACV VLP mRNA groups on individual days. Welch’s *t* test was performed when data met the assumption of normality; otherwise, the Mann–Whitney test was applied, * *p* < 0.05. Correlation between MACV and JUNV IgG titers in sera from mice immunized with MACV mRNA (**b**,**d**) or MACV VLP mRNA (**c**,**e**). Simple linear regression with R^2^ and *p* values indicated on each graph.

Previous studies employing a SARS-CoV-2 spike VLP mRNA reported not only an induction of higher levels IgG and neutralizing antibodies but also a stronger elicitation of cross-reactive antibodies. To address whether the MACV VLP mRNA vaccine would likewise increase the titers of cross-reactive antibodies, we established a Junin virus GPC-specific ELISA. JUNV is the most closely related NW arenavirus to MACV [55] with the Junin and Machupo virus GPC sharing 69% amino acid identity and 79.4% similarity. JUNV-specific IgG antibodies were measured in serum from MACV mRNA and VLP mRNA-vaccinated mice (Figure 4a). Before the boost, there were low and highly variable levels of JUNV IgG with many mice having no cross-reactivity. After the boost, the MACV VLP mRNA vaccine elicited modest JUNV antibody titers above 10^3^, while MACV mRNA-induced anti-JUNV GPC titers were highly variable and averaged around 10^15^. Two mice from the MACV mRNA group failed to develop any cross-reactive antibodies. In the MACV VLP mRNA group, there is a positive correlation between MACV IgG titers and JUNV titers. The same correlation is not observed in the MACV mRNA vaccination group, which is likely because there were animals that did not develop any JUNV-specific antibodies (Figure 4b–e). Overall, our results demonstrate that the MACV VLP mRNA vaccine elicited higher levels of JUNV-specific antibodies than the MACV mRNA vaccine.

To further characterize the breadth of the humoral immune response to vaccination, we measured cross-neutralizing antibodies via FRNT_50_. There were no cross-neutralizing antibodies present in the serum from any of the vaccinated mice, including after the boost.

### 3.5. T-Cell Response to Vaccination with MACV mRNA and MACV VLP mRNA

There is little published information on cellular responses to MACV vaccination. To characterize the cellular response to MACV mRNA vaccination, mice were vaccinated once or twice as described earlier. Mouse spleens were collected 10 days post-single vaccination or 10 days post-boost. Splenocytes were isolated and stimulated with peptide pools covering MACV GPC. The MACV glycoprotein precursor is translated as a polyprotein and cleaved by cellular proteases into the stable signal peptide, GP1, and GP2. GP1 is the surface glycoprotein responsible for receptor binding, and GP2 mediates viral fusion [19]. We evaluated separate peptide pools covering either MACV GP1 or GP2.

**Figure 5:**
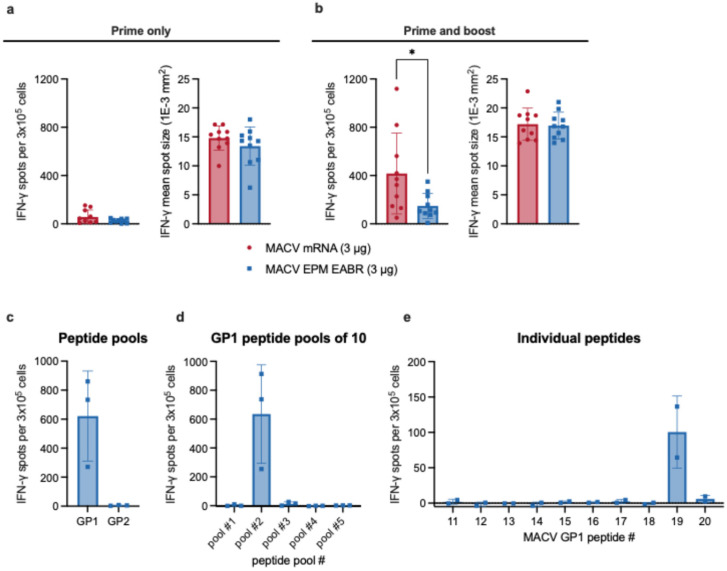
T-cell response to vaccination with MACV mRNA and MACV VLP mRNA. ELISpot assay data for MACV GP1-specific INF-γ responses of splenocytes from C57BL/6 mice (*n* = 10 per group) that were immunized with 1 dose (**a**) or 2 doses (**b**) of MACV mRNA (red circles) or MACV VLP mRNA (blue squares). Data are shown as spots per 3 × 10^5^ cells (left) and mean spot sizes (right). Statistical analyses were performed to compare the MACV mRNA and MACV VLP mRNA groups. Welch’s *t* test was performed, * *p* < 0.05. (**c**) Peptide pools of 15-mers spanning MACV GP1 (49 peptides) or GP2 (48 peptides) were used in an IFN-γ ELISPOT assay conducted 10 days post-immunization with two doses of MACV VLP mRNA (*n* = 3). (**d**) The 49 peptides spanning MACV GP1 were pooled into 4 groups of 10 peptides and one group of 9 peptides each; then, they were tested to narrow down immunogenic epitopes. (**e**) The individual peptides from pool #2 were then tested to determine immunogenic epitopes. For all graphs, individual points represent the mean of 2 technical replicates per animal, horizontal lines indicate the mean of the group, and error bars indicate ± standard deviation.

After stimulation, INF-γ secretion was measured via ELISpot to evaluate T-cell activation (Figure 5a,b). In preliminary experiments, we found that only the peptide pool from GP1 elicited a detectable T-cell response (Figure 5c). Therefore, all subsequent experiments utilized only the GP1 peptides. The T-cell response to a single dose of 3 μg of either MACV mRNA or MACV VLP mRNA was very low; the average IFN-γ spot counts were 56.7 and 24.8, respectively (Figure 5a). However, mice that received two doses of either vaccine had a robust IFN-γ response with significantly higher numbers of MACV GP reactive T cells in the MACV mRNA group (average spot count: 416.25) compared to the MACV VLP mRNA group (average spot count: 148.15) (Figure 5b).

To identify the MACV GP1 peptide(s) responsible for inducing the T-cell response in C57BL/6 mice, the pool of 49 GP1 15-mer peptides was broken down into pools of 10 peptides and analyzed for T-cell stimulation as above (Figure 5d). From this analysis, one 10-peptide pool was found to contain stimulatory activity. The individual peptides of this pool were then analyzed, and we were able to identify an immunodominant epitope from the MACV GP1 peptide pool (Figure 5e). Only one peptide (#19, LCMLNNSFYYMKGGV) induced a strong IFN-γ response.

Overall, the T-cell analysis identified a single immunodominant epitope in MACV GPC. Moreover, as was seen for neutralizing antibody responses, two doses of a MACV mRNA vaccine are required to induce a robust T-cell response. Additionally, the MACV VLP mRNA vaccine does not improve the T-cell response to vaccination, as was seen with the humoral response.

### 3.6. Comparing MACV mRNA and rVSV Vaccines

**Figure 6:**
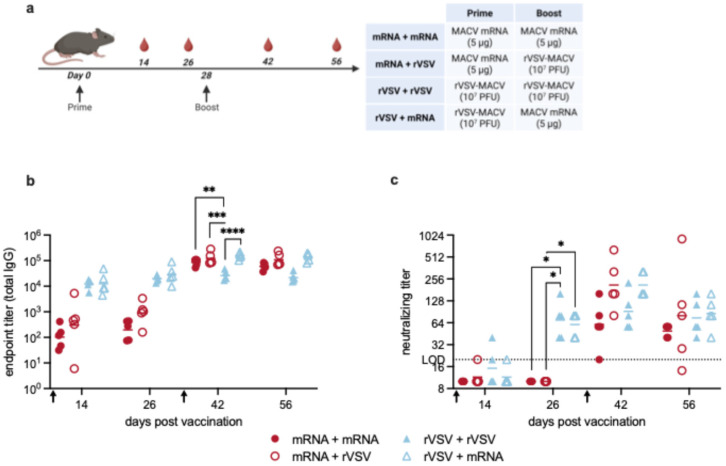
Humoral responses to rVSV-MACV and MACV mRNA vaccinations. (**a**) Experimental timeline for immunization in C57BL/6 mice. Mice (*n* = 5 per group) were vaccinated on day 0 and boosted on day 28, as indicated by black arrows. Red blood droplets indicate blood collection, which took place every 2 weeks for serum analysis of antibody titers. Vaccine groups are defined in the table. Total MACV-specific IgG titers (**b**) and neutralizing titers (**c**) in serum from vaccinated mice. Black arrows indicate the timing of prime and boost immunizations. Solid symbols indicate homologous prime-boost, and open symbols indicate heterologous prime-boost. Symbols represent individual animals, and the line indicates the geometric mean of the group. Each point represents the mean (ELISA) or geometric mean (FRNT) from 2–3 independent ELISA or FRNT analyses of each serum sample. LOD = limit of detection. Statistical analyses were performed to compare different vaccination groups on individual days. One-way ANOVA with Tukey’s multiple comparisons test was performed when data met the assumption of normality; otherwise, the Kruskal–Wallis with Dunn’s multiple comparisons test was applied, * *p* < 0.05, ** *p* < 0.01, *** *p* < 0.001, **** *p* < 0.0001. For clarity in panel (**b**), only comparisons on day 42 are shown; a summary of other comparisons is found in Table S1.

Next, we have developed a recombinant vesicular stomatitis virus (rVSV) vaccine encoding the MACV GPC (rVSV-MACV) in place of the VSV-G gene. We sought to compare the conventional MACV mRNA vaccine to this recombinant virus vectored construct. Mice were immunized with either 10^7^ plaque-forming units (PFUs) rVSV-MACV delivered intraperitoneally (i.p.) or 5 μg of MACV mRNA administered i.m. (Figure 6a). We anticipated a low replication of VSV-MACV in mice due to a receptor–glycoprotein mismatch. The mouse homologue of the main MACV receptor, transferrin receptor 1, does not bind MACV GPC efficiently [48], and the virus must enter through a less efficient pathway [49]. We chose the VSV vaccine dose to compensate for this and allow for VSV replication. To further address this issue and allow more efficient VSV replication, 0.5 μg/mouse of an anti-interferon–alpha/beta receptor (anti-IFNAR-1) blocking antibody was administered 1 day before and 1 day after vaccination. The route of administration for each vaccine in this paper was chosen based on the standard practice for that vaccine platform in mice to allow for an easier comparison to vaccines tested and published by other research groups. An analysis of antibody responses at days 14 and 26 revealed that the rVSV vaccinations (triangles, Figure 6b) induced high levels of IgG that were significantly (approximately 30-fold) higher than those seen with mRNA vaccination (circles, Figure 6b). The distinction between rVSV and mRNA vaccination was even more obvious when neutralizing antibody responses were examined. A single dose of rVSV-MACV elicited measurable neutralizing antibodies, whereas a single dose of MACV mRNA did not. Some of the rVSV-MACV-vaccinated mice developed neutralizing antibodies as soon as 14 days after vaccination, and all of them had neutralizing responses by day 26 prior to a boost (triangles, Figure 6c). In contrast, no mRNA-vaccinated animals mounted a consistent neutralizing response at these time points (circles, Figure 6c). The neutralizing antibody response on day 26 was significantly higher for the rVSV-vaccinated mice than for the mRNA-vaccinated mice (Figure 6c).

We next examined the effect of a booster immunization on the antibody response. With other viruses, heterologous vaccination strategies have been shown to increase antibody titers to levels beyond that of a single vaccine platform alone [45,56,57]. To test this idea for MACV, we boosted groups of mice, as shown in Figure 6a. A homologous (rVSV then rVSV, mRNA then mRNA) or heterologous (rVSV then mRNA, mRNA then rVSV) boost immunization was given at day 28. In mice primed with rVSV-MACV, the high titers induced by the prime titers were not boosted by a second dose of rVSV (closed triangles) but were boosted approximately 5-fold by a heterologous boost with MACV mRNA (open triangles, Figure 6b). By contrast, the low initial titers elicited by MACV mRNA were boosted using both a homologous second dose of mRNA (305.9-fold increase) or a heterologous rVSV-MACV boost (121.2-fold increase) (Figure 6b, open and closed circles). Neutralizing titers were also measured for each group. Following the boost, neutralizing titers for mice primed with mRNA were boosted to levels that were comparable to the titers elicited from rVSV vaccination. On day 42 and day 56, the differences between all vaccination groups were not statistically significant, although the heterologous boost groups (open shapes) had slightly higher neutralizing titers than the homologous boost groups (closed shapes) (Figure 6c).

Together, these data suggest that a heterologous vaccine strategy combining rVSV-MACV and the conventional MACV mRNA may elicit a more favorable immune response than either vaccine platform alone. rVSV followed by mRNA induces a faster antibody response (developing high IgG titers by day 14 and neutralizing antibodies by day 26) that after the boost reaches overall titers comparable to the homologous mRNA prime-boost vaccine regimen.

## 4. Discussion

MACV represents a significant public health threat. Despite research efforts, there are still no approved vaccines to combat this risk. In this paper, we developed and characterized the immune response to three types of MACV vaccines alone and in combination. The first is an mRNA-LNP vaccine encoding the full-length MACV GPC that we called MACV mRNA. The second was another mRNA-based vaccine; however, in this case, the MACV glycoprotein has sequences appended to the mRNA that result in MACV GPC expression on virus-like particles (VLPs); this is called MACV VLP mRNA. The third vaccine candidate is a replication-competent recombinant VSV (rVSV) vector expressing MACV GPC in place of VSV-G. All three vaccines elicited strong antibody responses; however, the magnitude of the response, the development of neutralizing antibodies, and the dosing requirements for an effective response were distinct.

A single dose of the conventional MACV mRNA vaccine elicited low IgG titers, no neutralizing antibodies, and low levels of MACV-specific T cells. This was somewhat unexpected, as a single dose of many mRNA vaccines encoding other viral glycoproteins can elicit robust immune responses [45,53,54,58]. A second vaccine dose was required to elicit a robust immune response, including high total IgG titers, low levels of neutralizing antibodies, and IFN-γ secreting T cells. The data from our study reinforce the idea that mRNA vaccine-elicited immune responses to different viral antigens are antigen-specific and that potential vaccine candidates for each pathogen must be evaluated empirically.

In an effort to improve the immune response to an mRNA-based vaccine, we engineered an mRNA construct that includes signals which cause the MACV GPC to bud from expressing cells as a virus-like particle (VLP) [44]. Multivalent particulate antigens, including virus-like particles (VLPs) and attenuated viruses, have demonstrated the ability to elicit robust and durable antibody responses after a single immunization [59]. Here, we show that MACV GPC with appended EPM and EABR sequences in the mRNA induces the budding of MACV VLPs, thus mimicking natural infection by expressing the glycoprotein on particles similar in size and shape to virions. Presentation of the MACV GPC antigen in particle form improved the humoral immune response elicited by our MACV VLP mRNA vaccine compared to the parent MACV mRNA. However, as was the case for the mRNA vaccine, the MACV VLP mRNA vaccine required a booster dose to elicit neutralizing antibodies.

In the study that established the VLP mRNA system with SARS-CoV-2 spike [44], the cross-neutralization of divergent SARS-CoV-2 viruses was observed, suggesting that the SARS-CoV-2 spike VLP mRNA vaccine induces an antibody response with increased breadth. We noted that the boosted MACV VLP mRNA vaccine also elicited an antibody response with increased breadth indicated by binding to and neutralizing two NW arenaviruses: MACV and JUNV. Previous studies have investigated cross-reactive antibodies between MACV and JUNV but have primarily focused on whether JUNV GPC-elicited antibodies could cross-react with MACV GPC [60,61]. While some studies have identified anti-JUNV antibodies that cross-react with MACV GPC to varying degrees [62], others point out that the structure of MACV GPC has a unique loop called loop 10 that interferes with the binding of cross-reactive antibodies [63]. The further investigation of increased breadth elicited by our MACV VLP mRNA, including testing cross-protection rather than just cross-reactivity, would further elucidate how broadly applicable the MACV mRNA vaccines may be against other NW arenaviruses.

Previous studies demonstrated that SARS-CoV-2 spike mRNA and SARS-CoV-2 spike VLP mRNA vaccines elicited similar levels of IFN-γ-secreting T cells [44]. However, in this paper, we observed that the T-cell response to MACV mRNA appeared higher than the T-cell response to MACV VLP mRNA. Future vaccine development must consider the type of immune response that is most important for each pathogen and balance the induction of humoral and cellular responses accordingly. Furthermore, T-cell responses to MACV remain largely unexplored. Although correlates of protection for MACV are not clearly defined, it is thought that neutralizing antibodies play a major role in protection, and most vaccine studies have focused on humoral immune responses. The further characterization of the T-cell response to MACV infection may elucidate other important information. In C57B/L6 mice, we noted a single MACV GP1 peptide (LCMLNNSFYYMKGGV) that induced a strong IFN-γ response. The Rankpep peptide prediction tool [64] identified the sequence CMLNNSFYY as the only strong MHC Db binding peptide, suggesting that this is an MACV GPC immunodominant epitope. This peptide should be useful in further studies in mice of T-cell responses induced by other MACV GPC vaccines.

Unlike the results with the two mRNA-LNP vaccines for MACV, a recombinant VSV vector expressing MACV GPC induced IgG and neutralizing antibodies as soon as 14 days after the administration of a single rVSV-MACV dose. The total anti-MACV GPC response was robust; however, the neutralizing titer was moderate compared to the IgG titer. Our results with rVSV-MACV align well with studies of the OW mammarenavirus Lassa in non-human primates where a single dose of the rVSVΔG-LASV-GPC vaccine candidate induced IgG and neutralizing antibodies by day 26 post-vaccination [65]. As was the case for MACV, the level of LASV-neutralizing antibodies was relatively low compared to the total IgG titer. This supports the idea that developing vaccines capable of inducing neutralizing antibodies to these mammarenaviral glycoproteins may be difficult.

We also investigated if heterologous vaccine strategies may be employed to elicit a more favorable immune response than either the rVSV or mRNA platforms alone. We demonstrate that a heterologous prime-boost vaccination regimen employing rVSV and then mRNA resulted in both rapid antibody development as well as the highest overall titers and neutralization. While immune characterization does not directly translate to protection, it is believed that a strong antibody response is important for NW mammarenavirus protection [21,22]. Future studies are required to evaluate the protective efficacy of each vaccine platform and vaccination protocol. Additionally, although we demonstrated that the boosted mRNA vaccine antibody and neutralization titers remained constant up to 118 days, it will be critical to evaluate the durability of the responses induced by the heterologous vaccination regimens.

Viral glycoproteins are dynamic structures that can exist in multiple configurations aside from the native conformation found on virions that is the target for neutralizing antibodies. For Lassa virus, neutralizing antibodies against GPC principally target quaternary epitopes displayed only on the metastable, pre-fusion conformation of GPC [65]. It is possible that the reason MACV mRNA immunization produces significant IgG titers, but very low neutralization is that the MACV mRNA expression results in some portion of the antigen in a non-native or post-fusion conformation. The recombinant MACV GPC protein produced for the ELISA analysis could also contain a portion of this non-native structure, while the target for neutralization on the virion is only the native, pre-fusion conformation. Recently, engineered versions of viral glycoproteins have been designed to lock the protein in the native state and thus maintain the highly immunogenic, pre-fusion oligomeric structure for high vaccine efficacy. mRNA vaccines for respiratory syncytial virus and SARS-CoV-2 are examples where the expression of stabilized viral glycoproteins proteins are important for efficacy [66,67]. Similar mutations have been engineered into the OW mammarenavirus Lassa virus GPC, and it has been suggested that these may enhance the antibody response to Lassa [68]. Stabilizing mutations in the MACV and JUNV GPC proteins that lock the proteins in the native conformation have recently been described [69].

Beyond immunogenicity, there are practical concerns that affect the real-world effectiveness of any given vaccine platform, and those factors must be considered when choosing a vaccine type or immunization strategy. Our results show that a single dose of rVSV-MACV induces a rapid humoral immune response, including neutralizing antibodies. The development of a one-dose vaccine that does not require a follow-up booster is important for pathogens like MACV that are found in rural or under-resourced areas. The FDA-approved rVSV Ebola virus vaccine (Ervebo) is an example of an rVSV vaccine that successfully induces a protective response after a single dose and was deployed in under-resourced areas in West Africa during the 2018–2020 Ebola virus outbreaks [28,32,70]. In contrast, mRNA vaccines have not been as widely deployed in under-resourced settings, and current versions require a cold chain that is often unavailable. Our results indicate that at least two doses of a MACV mRNA vaccine would be needed in humans.

There may be different scenarios where different vaccine platforms may be most effective. For example, the use of mRNA vaccines may make sense for healthcare workers at risk of nosocomial MACV infection. In this case, there would likely be less of a barrier to receiving two doses of vaccines. We found that homologous mRNA prime-boost or rVSV prime- mRNA boost combinations induced the highest levels of total and neutralizing antibodies. In contrast, the use of rVSV-MACV vaccines may be a more favorable option in other scenarios such as outbreaks, when more immediate protection is needed, or in rural areas with healthcare infrastructure that would limit the availability of vaccine and booster doses.

Overall, the data presented here highlight the potential of three vaccine platforms for MACV. Further studies into the protective efficacy of MACV mRNA, MACV VLP mRNA, and rVSV-MACV will help in efforts to develop safe and effective vaccines for MACV.

## 5. Conclusions

In this paper, we present and compare three vaccine candidates for MACV: rVSV-MACV, MACV mRNA, and MACV VLP mRNA. We conclude that two doses of MACV mRNA or MACV VLP mRNA are required to induce strong humoral and cellular responses in mice. We also conclude that heterologous mRNA and rVSV prime-boost regimens induced higher levels of MACV GPC-specific IgG and neutralizing titers than homologous prime-boost strategies. These results highlight differences in the immune response to different vaccine platforms for MACV and can inform future vaccine development for NW arenaviruses.

## Data Availability

The raw data supporting the conclusions of this article will be made available by the authors on request.

## Supplementary Materials

The following supporting information can be downloaded at https://www.mdpi.com/article/10.3390/vaccines14040315/s1, Table S1: Statistical analyses of humoral responses to rVSV-MACV and MACV mRNA vaccinations; Figure S1: Western blot showing the in vitro expression of transfected MACV mRNA; Figure S2: top panel; Figure S3: bottom panel.
