# Supplementary material for: Comparison of Vaccine Platforms for Machupo Virus
Source: Vaccines (Basel). 2026 Mar 31;14(4):315. doi: 10.3390/vaccines14040315 (PMC13120303; doi:10.3390/vaccines14040315)
Supplement: Supplementary file 1 [file vaccines-14-00315-s001.zip › Figures S1-3.pdf]

Figure S1 Western blot showing the in vitro expression of transfected MACV mRNA.

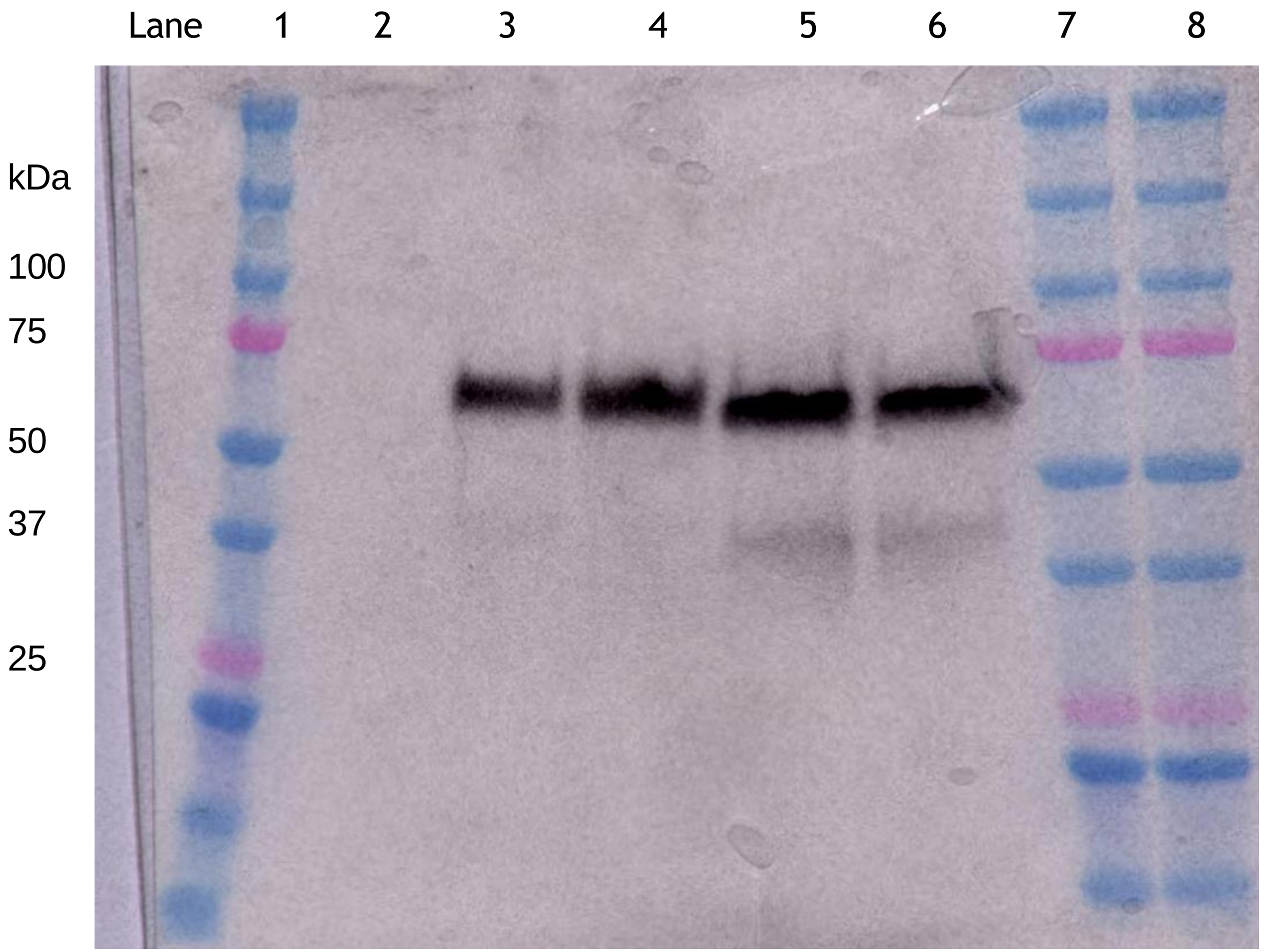

Original, uncropped, and unadjusted image used in **Figure 1a**. Lanes 2-4 are represented in the figure. Lanes 5-8 include samples from a different experiment run concurrently that are not relevant to this figure.

Lane 1: ladder
Lane 2: cell lysate from cells transfected with Luciferase mRNA
Lane 3: cell lysate from cells transfected with MACV GPC mRNA
Lane 4: cell lysate from cells transfected with pCG1 MACV GPC (DNA plasmid)

Figure S2- top panel

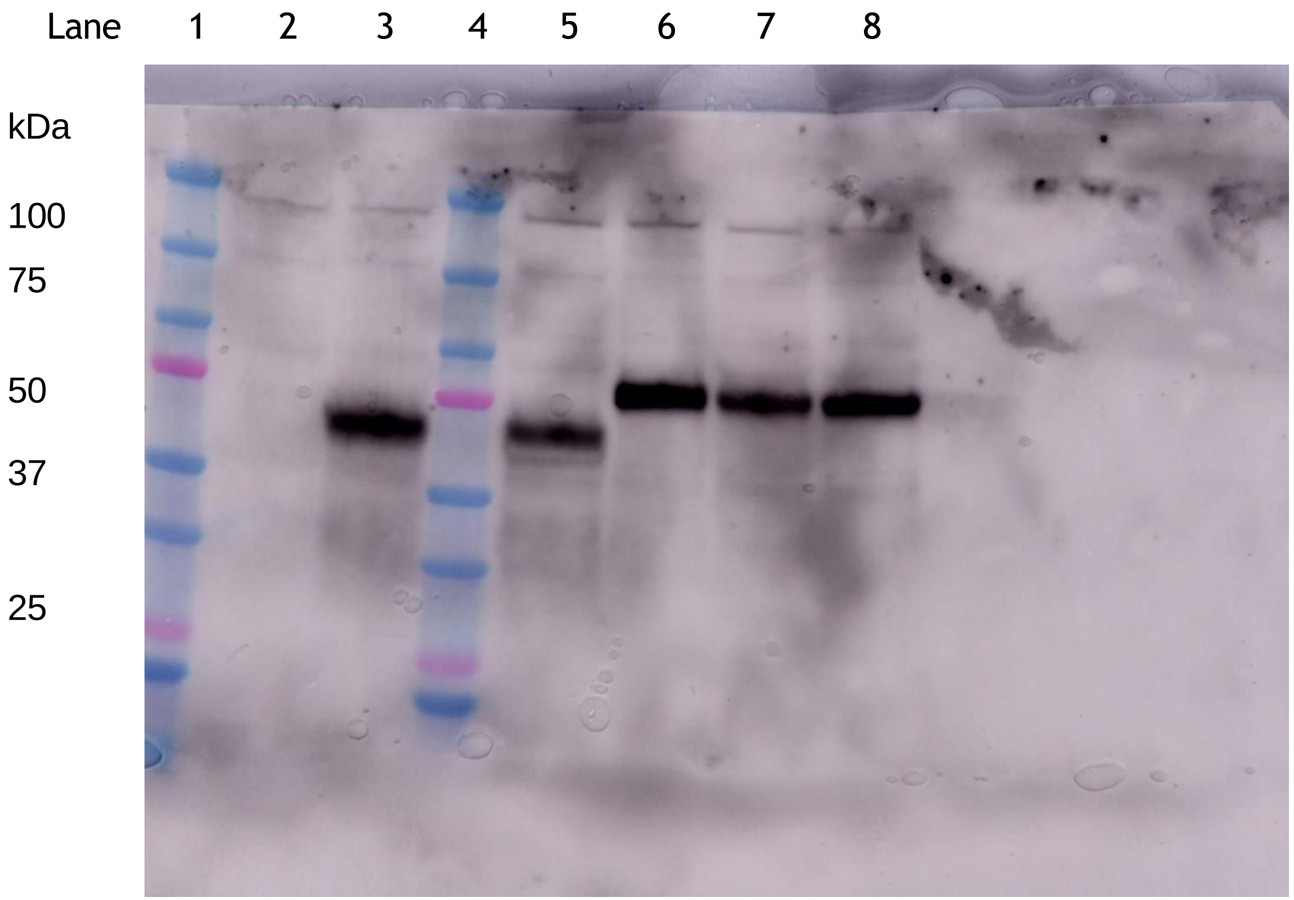

Original, uncropped, and unadjusted image used in **Figure 3b, top panel**. Lanes 5-8 are represented in the figure. Other lanes include samples from a different experiment run concurrently that are not relevant to this figure.

Lane 4: ladder
Lane 5: cell lysate from cells transfected with pCG1 MACV GPC
Lane 6: cell lysate from cells transfected with MACV GPC (full-length) + EPM EABR
Lane 7: cell lysate from cells transfected with MACV Δ13 + EPM EABR
Lane 8: cell lysate from cells transfected with MACV Δ25 + EPM EABR

Figure S3- bottom panel

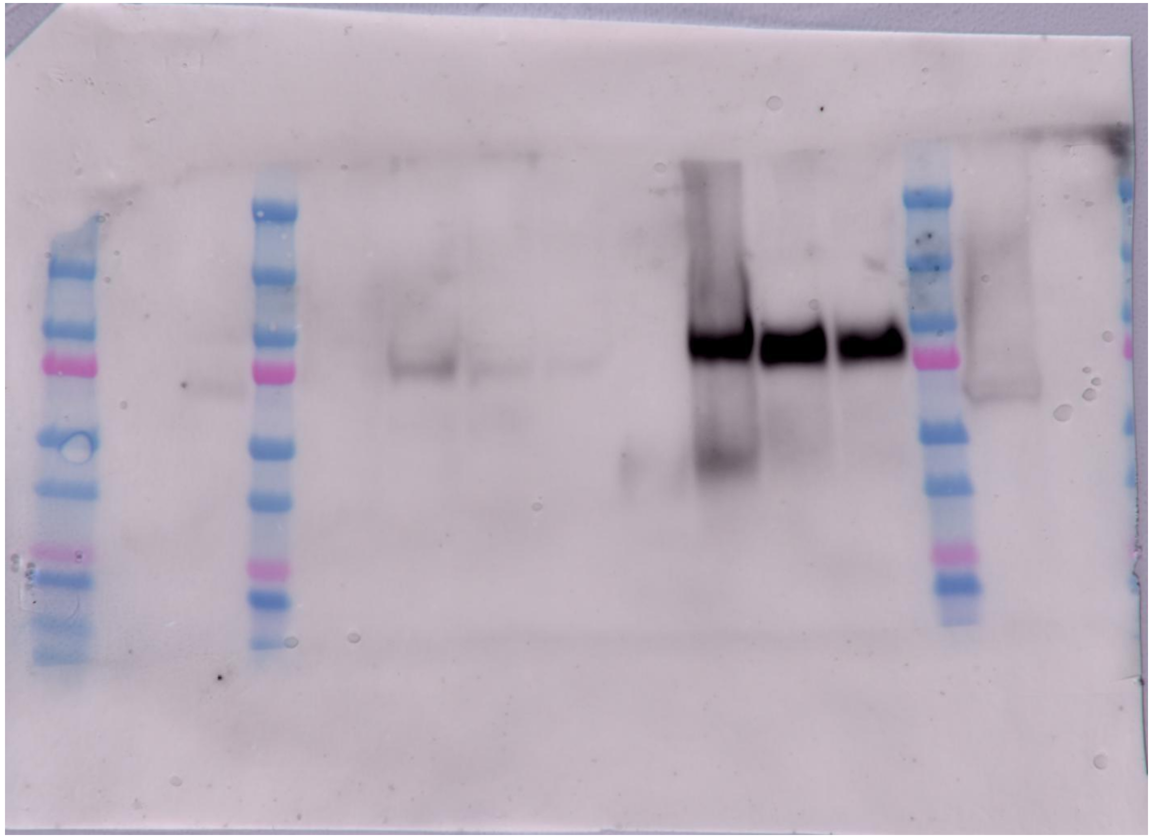

Original, uncropped, and unadjusted image used in **Figure 3b, bottom panel**. Lanes 9-12 are represented in the figure. Other lanes include samples from a different experiment run concurrently that are not relevant to this figure.

Lane 9: cell lysate from cells transfected with pCG1 MACV GPC
Lane 2: cell lysate from cells transfected with MACV GPC (full-length) + EPM EABR
Lane 3: cell lysate from cells transfected with MACV Δ13 + EPM EABR
Lane 4: cell lysate from cells transfected with MACV Δ25 + EPM EABR
Lane 13: ladder
