# Supplementary material for: Comparison of Vaccine Platforms for Machupo Virus
Source: Vaccines (Basel). 2026 Mar 31;14(4):315. doi: 10.3390/vaccines14040315 (PMC13120303; doi:10.3390/vaccines14040315)
Supplement: Supplementary file 1 [file vaccines-14-00315-s001.zip › Table S1.pdf]

Table S1

Figure	Time Point	Groups Compared	Summary	Adjusted P Value
Figure 6 b	day 14	mRNA + mRNA vs. mRNA + VSV	ns	0.637
		mRNA + mRNA vs. VSV + VSV	***	0.0004
		mRNA + mRNA vs. VSV + mRNA	***	0.0004
		mRNA + VSV vs. VSV + VSV	**	0.0041
		mRNA + VSV vs. VSV + mRNA	**	0.0039
		VSV + VSV vs. VSV + mRNA	ns	>0.9999
	day 26	mRNA + mRNA vs. mRNA + VSV	*	0.0453
		mRNA + mRNA vs. VSV + VSV	****	<0.0001
		mRNA + mRNA vs. VSV + mRNA	****	<0.0001
		mRNA + VSV vs. VSV + VSV	***	0.0001
		mRNA + VSV vs. VSV + mRNA	****	<0.0001
		VSV + VSV vs. VSV + mRNA	ns	0.8996
	day 42	mRNA + mRNA vs. mRNA + VSV	ns	0.5501
		mRNA + mRNA vs. VSV + VSV	**	0.0016
		mRNA + mRNA vs. VSV + mRNA	ns	0.1448
		mRNA + VSV vs. VSV + VSV	***	0.0001
		mRNA + VSV vs. VSV + mRNA	ns	0.7868
		VSV + VSV vs. VSV + mRNA	****	<0.0001
	day 56	mRNA + mRNA vs. mRNA + VSV	ns	0.1587
		mRNA + mRNA vs. VSV + VSV	**	0.0081
		mRNA + mRNA vs. VSV + mRNA	*	0.0339
		mRNA + VSV vs. VSV + VSV	****	<0.0001
		mRNA + VSV vs. VSV + mRNA	ns	0.836
		VSV + VSV vs. VSV + mRNA	****	<0.0001
Fig 6 c	day 14	mRNA + mRNA vs. mRNA + VSV	ns	>0.9999
		mRNA + mRNA vs. VSV + VSV	ns	0.6436
		mRNA + mRNA vs. VSV + mRNA	ns	>0.9999
		mRNA + VSV vs. VSV + VSV	ns	>0.9999
		mRNA + VSV vs. VSV + mRNA	ns	>0.9999
		VSV + VSV vs. VSV + mRNA	ns	>0.9999
	day 26	mRNA + mRNA vs. mRNA + VSV	ns	>0.9999
		mRNA + mRNA vs. VSV + VSV	*	0.032
		mRNA + mRNA vs. VSV + mRNA	ns	0.0535
		mRNA + VSV vs. VSV + VSV	*	0.0188
		mRNA + VSV vs. VSV + mRNA	*	0.0332
		VSV + VSV vs. VSV + mRNA	ns	>0.9999
	day 42	mRNA + mRNA vs. mRNA + VSV	ns	0.1226
		mRNA + mRNA vs. VSV + VSV	ns	>0.9999
		mRNA + mRNA vs. VSV + mRNA	ns	0.0726
		mRNA + VSV vs. VSV + VSV	ns	0.545

		mRNA + VSV vs. VSV + mRNA	ns	>0.9999
		VSV + VSV vs. VSV + mRNA	ns	0.359
	day 56	mRNA + mRNA vs. mRNA + VSV	ns	>0.9999
		mRNA + mRNA vs. VSV + VSV	ns	>0.9999
		mRNA + mRNA vs. VSV + mRNA	ns	0.6594
		mRNA + VSV vs. VSV + VSV	ns	>0.9999
		mRNA + VSV vs. VSV + mRNA	ns	>0.9999
		VSV + VSV vs. VSV + mRNA	ns	>0.9999

Table S1: Statistical analyses of humoral responses to rVSV-MACV and MACV mRNA vaccinations. Statistical analyses were performed to compare different vaccination groups on individual days. One-way ANOVA with Tukey's multiple comparisons test was performed when data met the assumption of normality; otherwise, the Kruskal–Wallis with Dunn's multiple comparisons test was applied, ns = not significant, *p < 0.05, **p < 0.01, ***p < 0.001 , ****p < 0.0001.
